# Respiratory and Psychophysical Sequelae Among Patients With COVID-19 Four Months After Hospital Discharge

**DOI:** 10.1001/jamanetworkopen.2020.36142

**Published:** 2021-01-27

**Authors:** Mattia Bellan, Daniele Soddu, Piero Emilio Balbo, Alessio Baricich, Patrizia Zeppegno, Gian Carlo Avanzi, Giulia Baldon, Giuseppe Bartolomei, Marco Battaglia, Sofia Battistini, Valeria Binda, Margherita Borg, Vincenzo Cantaluppi, Luigi Mario Castello, Elisa Clivati, Carlo Cisari, Martina Costanzo, Alessandro Croce, Daria Cuneo, Carla De Benedittis, Simona De Vecchi, Alessandro Feggi, Martina Gai, Eleonora Gambaro, Eleonora Gattoni, Carla Gramaglia, Leonardo Grisafi, Chiara Guerriero, Eyal Hayden, Amalia Jona, Marco Invernizzi, Luca Lorenzini, Lucia Loreti, Maria Martelli, Paolo Marzullo, Erica Matino, Antonio Panero, Elena Parachini, Filippo Patrucco, Giuseppe Patti, Alice Pirovano, Pierluigi Prosperini, Riccardo Quaglino, Cristina Rigamonti, Pier Paolo Sainaghi, Camilla Vecchi, Erika Zecca, Mario Pirisi

**Affiliations:** 1 Department of Translational Medicine, Università del Piemonte Orientale, Novara, Italy; 2 Azienda Ospedaliero–Universitaria Maggiore della Carità, Novara, Italy

## Abstract

**Importance:**

Although plenty of data exist regarding clinical manifestations, course, case fatality rate, and risk factors associated with mortality in severe coronavirus disease 2019 (COVID-19), long-term respiratory and functional sequelae in survivors of COVID-19 are unknown.

**Objective:**

To evaluate the prevalence of lung function anomalies, exercise function impairment, and psychological sequelae among patients hospitalized for COVID-19, 4 months after discharge.

**Design, Setting, and Participants:**

This prospective cohort study at an academic hospital in Northern Italy was conducted among a consecutive series of patients aged 18 years and older (or their caregivers) who had received a confirmed diagnosis of severe acute respiratory coronavirus 2 (SARS-CoV-2) infection severe enough to require hospital admission from March 1 to June 29, 2020. SARS-CoV-2 infection was confirmed via reverse transcription–polymerase chain reaction testing, bronchial swab, serological testing, or suggestive computed tomography results.

**Exposure:**

Severe COVID-19 requiring hospitalization.

**Main Outcomes and Measures:**

The primary outcome of the study was to describe the proportion of patients with a diffusing lung capacity for carbon monoxide (D_lco_) less than 80% of expected value. Secondary outcomes included proportion of patients with severe lung function impairment (defined as D_lco_ <60% expected value); proportion of patients with posttraumatic stress symptoms (measured using the Impact of Event Scale–Revised total score); proportion of patients with functional impairment (assessed using the Short Physical Performance Battery [SPPB] score and 2-minute walking test); and identification of factors associated with D_lco_ reduction and psychological or functional sequelae.

**Results:**

Among 767 patients hospitalized for severe COVID-19, 494 (64.4%) refused to participate, and 35 (4.6%) died during follow-up. A total of 238 patients (31.0%) (median [interquartile range] age, 61 [50-71] years; 142 [59.7%] men; median [interquartile range] comorbidities, 2 [1-3]) consented to participate to the study. Of these, 219 patients were able to complete both pulmonary function tests and D_lco_ measurement. D_lco_ was reduced to less than 80% of the estimated value in 113 patients (51.6%) and less than 60% in 34 patients (15.5%). The SPPB score was suggested limited mobility (score <11) in 53 patients (22.3%). Patients with SPPB scores within reference range underwent a 2-minute walk test, which was outside reference ranges of expected performance for age and sex in 75 patients (40.5%); thus, a total of 128 patients (53.8%) had functional impairment. Posttraumatic stress symptoms were reported in a total of 41 patients (17.2%).

**Conclusions and Relevance:**

These findings suggest that at 4 months after discharge, respiratory, physical, and psychological sequelae were common among patients who had been hospitalized for COVID-19.

## Key Points

**Question:**

What respiratory, functional, and psychological sequalae are associated with recovery from coronavirus disease 2019 (COVID-19)?

**Findings:**

In this cohort study of 238 patients with COVID-19 hospitalized in an academic hospital in Northern Italy, more than half of participants had a significant reduction of diffusing lung capacity for carbon monoxide or measurable functional impairment and approximately one-fifth of patients had symptoms of posttraumatic stress 4 months after discharge.

**Meaning:**

These findings suggest that despite virological recovery, a sizable proportion of patients with COVID-19 experienced respiratory, functional, or psychological sequelae months after hospital discharge.

## Teaser

This cohort study examines prevalence and risk factors associated with lung function or physical impairment or posttraumatic stress symptoms among survivors of severe coronavirus disease 2019 (COVID-19).

## Introduction

Severe acute respiratory syndrome coronavirus 2 (SARS-CoV-2) infection can be completely asymptomatic or, conversely, can cause coronavirus disease 2019 (COVID-19), for which the clinical outcomes range from mild upper airways symptoms to a severe disease with respiratory failure and a high fatality rate.^1^ As of November 2020, more than 60 million people have been infected with SARS-CoV-2 worldwide, and more than 1.4 million people have died.^2^

Since the beginning of the COVID-19 pandemic, many researchers focused attention on clinical features and prognosis of the acute phase of SARS-CoV-2 infection.^3,4^ As a consequence, we are now far more able to estimate prognoses and optimize clinical treatment of patients with COVID-19 compared with the beginning of the pandemic.^5^

In contrast, the type and severity of respiratory or functional sequelae COVID-19 are unknown. While COVID-19 is a systemic disease,^6^ the lungs are most commonly affected, with histopathological findings that may include diffuse alveolar epithelium destruction, capillary damage or bleeding, hyaline membrane formation, alveolar septal fibrous proliferation, and pulmonary consolidation.^7^ As a consequence, the diffusion capacity of the lung for carbon monoxide (D_lco_) is commonly altered in patients who recover from COVID-19,^8^ similarly to SARS and Middle East respiratory syndrome (MERS), illnesses that are associated with an impairment of lung function lasting months to years.^9,10^ Impairment in exercise capacity often parallels D_lco_ reduction: patients who recover from SARS pneumonia have been found to have 6-minute walking test and 36-item Short Form General Health Survey scores persistently lower than the general population.^9^ Moreover, the functional impairment associated with COVID-19 may also be associated with adverse psychological outcomes. A multidisciplinary approach investigating the functional and psychological aspects associated with COVID-19 may be more effective in disclosing potential sequelae associated with COVID-19. In this prospective cohort study, we aimed to investigate prevalence and clinical associations of functional and psychological impairment 4 months after recovery from COVID-19.

## Methods

This cohort study was approved by the Comitato Etico Interaziendale Novara ethical committee. All participants provided written informed consent. This study was conducted in strict accordance with the principles of the Declaration of Helsinki^11^ and reported following the Strengthening the Reporting of Observational Studies in Epidemiology (STROBE) reporting guideline for cohort studies.

### Study Population

We contacted 767 consecutive patients (or their caregivers) aged 18 years or older who were discharged between March 1 and June 29, 2020, from the Azienda Ospedaliero–Universitaria Maggiore della Carità university hospital in Novara, Italy, where they had been admitted for COVID-19. Patients were contacted by telephone, and telephone follow-up was performed 3 to 4 months after discharge. A total of 35 patients (4.6%) died after discharge. Of the remaining patients, 494 (64.4%) declined participation (Figure). Among 238 patients who agreed to participate, 232 participants (97.5%) had diagnosis of COVID-19 confirmed during their hospital stay by reverse-transcription–polymerase chain reaction (RT-PCR) of a nasopharyngeal swab; in 1 participants whose RT-PCR test was negative for SARS-CoV-2, the diagnosis was confirmed by bronchoalveolar lavage. The remaining 5 participants were diagnosed according to a combination of serological tests positive for SARS-CoV-2 antibodies and suggestive computed tomography.

**Figure.**
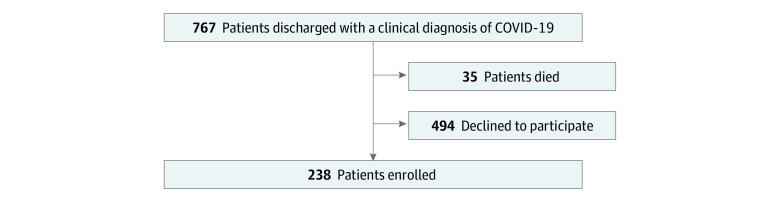
Flowchart of the Study Population COVID-19 indicate coronavirus disease 2019.

An electronic case report form was generated using the Research Electronic Data Capture software (Vanderbilt University) to collect clinical data following pseudonymization. Data entry was performed by clinicians involved in the treatment of patients with COVID-19; inpatient clinical data were retrospectively collected from clinical charts.

We collected data on patients’ demographic characteristics and regular medication use, symptoms at COVID-19 diagnosis and complications during the hospital stay (retrospectively documented), and type and number of comorbidities, including hypertension, type 2 diabetes, dyslipidemia, chronic obstructive pulmonary disease (COPD), obesity, inflammatory bowel disease, chronic liver disease, autoimmune disease, hematological diseases, coronary artery disease (CAD), atrial fibrillation and other structural or arrythmogenic heart disease, endocrine diseases, chronic kidney disease (CKD), previous stroke or venous thromboembolism, anxiety and depression, or active malignant neoplasm. We additionally collected data on patients’ symptoms at follow-up, including fever, cough, dyspnea, ageusia, anosmia, diarrhea, arthralgia, myalgia, chest pain, sore throat, headache, and perception of reduced tolerance to physical activity compared with before they contracted COVID-19.

### Pulmonary Function Tests

All patients underwent standard pulmonary function testing (PFT) with a Quark PFT with X9 pneumotach (COSMED) for forced expiratory volume in 1 second (FEV_1_), vital capacity, forced vital capacity (FVC), D_lco_, D_lco_ constant, and total lung capacity. D_lco_ and total lung capacity were determined by the single-breath co technique. The hemoglobin value was evaluated before PFT to apply the appropriate correction to D_lco_.

The spirometer underwent calibration the day the test was performed, and barometric pressure and temperature were simultaneously recorded. A trained technician coached the patient, while a pulmonologist (E. C., E. P., or F. P.) was responsible for test validation and interpretation based on the 2005 the American Thoracic Society and European Respiratory Society statements.^12,13^ Briefly, the following safety measures were adopted: in a dedicated room, a dedicated spirometer was used to avoid cross-infection of patients not included in this program. The technician (and the pulmonologist, if needed) used full PPE (ie, face mask, N95 respirator, gown, and gloves). To avoid cross-infections between patients included in the program, a mouthpiece with an antimicrobial filter was used and changed for every patient. At the end of each day, the room underwent disinfection.

### Physical Performance Tests

We assessed the patients’ physical performance with the Short Physical Performance Battery (SPPB), which includes balance assessment in standing position, walking speed for 4 m, and standing up from a chair with 5 repetitions. This test allows hierarchizing patients according to their functional status with a good predictivity on the disability level in daily activities. A score greater than 10 is the expected value for healthy individuals.^14,15^

However, it should be noted that SPPB may not distinguish performance level in high-functioning patients.^16^ To improve the sensitivity of functional impairment detection, patients with SPPB scores greater than 10 were tested with the 2-minute walk test to evaluate the residual aerobic capacity; the 2-minute walk test score was compared with reference data for an age- and sex-matched population.^17,18,19^

### Psychological Symptoms Tests

We assessed the presence of posttraumatic stress (PTS) symptoms by administering the Impact of Event Scale–Revised (IES-R),^20^ a 15-item self-rated 4-point scale based on how often an event has occurred in the past 7 days (0 indicates not at all; 1, rarely; 3, sometimes; 5, often). All IES-R items are anchored to a specific stressor. Besides the IES total subjective stress score, 2 subscales were identified. One subscale measured intrusive symptoms, including intrusive thoughts, nightmares, and intrusive feelings and imagery, using 7 items, with scores ranging from 0 to 35; the other subscale measured avoidance symptoms, such as numbing of responsiveness and avoidance of feelings, situations, or ideas, using 8 items, with scores ranging from 0 to 40.

### Statistical Analysis

Data were analyzed using the Stata statistical software version 15.1 (StataCorp). Normality was assessed by Shapiro-Wilk test. The measures of centrality and dispersion chosen for continuous variables were medians and interquartile ranges (IQRs); comparisons between groups for these variables were performed using the Mann-Whitney test. Categorical variables, whenever dichotomous or nominal, were reported as frequencies and percentages and analyzed through the Pearson χ^2^, Cochran-Armitage test, or Fisher exact test, as appropriate. The primary end point was the proportion of patients with a D_lco_ less than 80% of expected. The study was sufficiently powered to detect as statistically significant a 0.12 increase in the proportion of patients with D_lco_ less than 80% of expected among survivors of COVID-19 compared with that observed in a reference population^21^ (0.30 vs 0.18, respectively), with an α = .005. Secondary end points were prevalence of a more severe respiratory impairment (defined as D_lco_ <60% of expected), potentially associated with a higher risk of pulmonary fibrosis; factors associated with a D_lco_ less than 80% or less than 60% of expected; prevalence and factors associated with functional impairment (defined as SBBP score <11 or SBBP score ≥11 in presence of a 2-minute walk test score outside of age- and sex-matched reference range); and prevalence and factors associated with moderate to severe PTS symptoms.

To identify the associations with the different end points used, we conducted a univariate analysis, including the comorbidities with a biological plausible correlation with disease sequelae, age, sex, smoking status, intensive care unit (ICU) admission during hospital stay, number of comorbidities, and modality of oxygen delivery during hospital stay. *P* values were 2-sided, and statistical significance was set at *P* = .05. All associations with *P* < .20 were then included in logistic regression models.

## Results

Among 238 patients included in analysis, the median (IQR) age was 61 (50-71) years, and 142 (59.7%) were men. The main characteristics of participants are listed in Table 1. During hospital stays, 66 patients (27.7%) did not require supplementary oxygen, 102 patients (42.9%) received oxygen via nasal cannulae or Venturi masks, 49 patients (20.6%) required noninvasive ventilation, and 21 patients (8.8%) underwent mechanical ventilation. A total of 28 patients (11.8%) were admitted to an ICU, with a median (IQR) stay of 8.5 (5.5-20.5) days.

**Table 1.** Demographic Characteristics and Comorbidities of the Study Population and Symptoms of COVID-19 at Baseline and at Follow-up

Characteristic	Patients, No. (%)
Age, median (IQR), y	61 (50-71)
Medications used regularly, median (IQR), No.	2 (1-5)
Smoking status	
Never	139 (58.4)
Former	74 (31.1)
Current	25 (10.5)
Pack-years, median (IQR)	15 (7.25-36)
Comorbidities	
Total, median (IQR), No.	2 (1-3)
Arterial hypertension	98 (41.2)
Diabetes	36 (15.1)
Dyslipidemia	20 (8.4)
COPD	14 (5.8)
Obesity	25 (10.5)
IBD	4 (1.7)
Chronic liver disease	7 (2.9)
Autoimmune disease	5 (2.1)
Hematological disease	15 (6.3)
Ischemic cardiopathy	22 (9.2)
Atrial fibrillation	17 (7.1)
Other structural heart disease	4 (1.7)
Other arrhythmogenic heart disease	6 (2.5)
Endocrinological disease	26 (10.9)
Chronic kidney disease	15 (6.3)
Stroke or TIA	10 (4.2)
VTE	6 (2.5)
Anxiety and depression	11 (4.6)
Active malignant neoplasm	24 (10.1)
COVID-19 Symptoms	
Fever	
Acute phase	215 (90.3)
At follow-up	0
Cough	
Acute phase	132 (55.5)
At follow-up	6 (2.5)
Dyspnea	
Acute phase	129 (54.2)
At follow-up	13 (5.5)
Ageusia	
Acute phase	70 (29.4)
At follow-up	12 (5.0)
Anosmia	
Acute phase	63 (26.5)
At follow-up	11 (4.6)
Diarrhea	
Acute phase	54 (22.7)
At follow-up	3 (1.3)
Arthralgia	
Acute phase	46 (19.3)
At follow-up	14 (5.9)
Myalgia	
Acute phase	45 (18.9)
At follow-up	14 (5.9)
Chest pain	
Acute phase	2 (0.8)
At follow-up	1 (0.4)
Sore throat	
Acute phase	1 (0.4)
At follow-up	0
Headache	
Acute phase	1 (0.4)
At follow-up	0

Abbreviations: COPD, chronic obstructive pulmonary disease; COVID-19, coronavirus disease 2019; IBD, inflammatory bowel diseases; IQR, interquartile range; TIA, transient ischemic attack; VTE, venous thromboembolism.

Fever, cough, and dyspnea were the most commonly reported symptoms during the acute phase but largely remitted in the following months. However, 13 patients (5.5%) still reported dyspnea at 4 months after discharge (Table 1). At 4 months, 12 patients (5.0%) still experienced ageusia and 11 patients (4.6%) still experienced anosmia. Additionally, 14 patients (5.9%) reported still experiencing arthralgia at follow-up, and 14 patients (5.9%) reported still experiencing myalgia.

### PFTs

A total of 14 patients were not able to complete PFTs. Among the remaining 224 patients, the median (IQR) FEV_1_ was 101% (91.5%-112%) of expected and the median (IQR) FVC was 98.5% (90%-109%) of expected. Five more patients were not able to complete the assessment of the D_lco_, which was therefore measured in 219 patients. The median (IQR) D_lco_ was 79% (69%-89%) of expected. D_lco_ was less than 80% of expected in 113 patients (51.6%); more severe impairment (ie, D_lco_ <60% of expected) was observed in 34 patients (15.5%). The results of the univariate analysis of factors associated with impaired D_lco_ are reported in eTable 1 and eTable 2 in the Supplement. In logistic regression analysis, risk factors associated with D_lco_ less than 80% of expected at follow-up included female sex (odds ratio [OR], 4.33 [95% CI, 2.25-8.33]; *P* < .001), CKD (OR, 10.12 [95% CI, 2.00-51.05]; *P* = .005), and the modality of oxygen delivery during hospital stay (OR, 1.68 [95% CI, 1.08-2.61]; *P* = .02). Risk factors associated with D_lco_ less than 60% at follow-up were female sex (OR, 2.70 [95% CI, 1.11-6.55]; *P* = .03), COPD (OR, 5.52 [95% CI, 1.32-23.08]; *P* = .02), and ICU admission during hospital stay (OR, 5.76 [95% CI, 1.37-24.25]; *P* = .02) (Table 2).

**Table 2.** Logistic Regression Analysis of Risk Factors for D_lco_ Impairment

Outcome	OR (95% CI)	*P* value
**D_lco_ <80%**		
Female sex	4.33 (2.25-8.33)	<.001
Age	1.01 (0.99-1.04)	.17
Atrial fibrillation	1.48 (0.41-5.37)	.55
CKD	10.12 (2.00-51.05)	.005
ICU admission	1.32 (0.39-4.42)	.65
Modality of oxygen delivery	1.68 (1.08-2.61)	.02
COPD	2.20 (0.57-8.48)	.25
Smoking status	1.19 (0.76-1.84)	.45
D_lco_ **<60%**		
Female sex	2.70 (1.11-6.55)	.03
Age	1.00 (0.97-1.04)	.70
No. of comorbidities	1.18 (0.65-2.15)	.59
CKD	4.75 (1.19-19.00)	.03
Diabetes	2.17 (0.68-6.92)	.19
ICU admission	5.76 (1.37-24.25)	.02
Modality of oxygen delivery	1.55 (0.82-2.94)	.18
COPD	5.52 (1.32-23.08)	.02
Smoking status	0.98 (0.52-1.87)	.96

Abbreviations: CKD, chronic kidney disease; COPD, chronic obstructive pulmonary disease; D_lco_, diffusing lung capacity for carbon monoxide; ICU, intensive care unit; OR, odds ratio.

### Physical Performance Evaluation

With regard to physical function, 53 patients (22.3%) were found to have limited mobility based on SPPB test results. All other patients underwent a 2-minute walk test, which revealed a subtler impairment in 75 patients (31.5%). By this method, we identified 128 patients (53.8%) with some degree of functional impairment. The results of univariate analysis are reported in eTable 3 in the Supplement, and logistic regression analysis results are reported in Table 3. COPD was associated with an increased risk of physical impairment (OR, 12.70 [95% CI, 1.41-114.85]; *P* = .02), and higher D_lco_ was associated with decreased risk of physical impairment (OR, 0.96 [95% CI, 0.94-0.98]; *P* < .001).

**Table 3.** Logistic Regression Analysis of Factors Associated With Functional Impairment

Outcome	OR (95% CI)	*P* value
Functional impairment^a^		
Sex	1.22 (0.61-2.44)	.57
Age	0.99 (0.97-1.02)	.85
No. of comorbidities	1.51 (0.96-2.37)	.07
ICU admission	1.47 (0.42-5.06)	.54
Modality of oxygen delivery	1.10 (0.69-1.74)	.70
Diabetes	0.95 (0.35-2.60)	.92
Obesity	2.70 (0.81-9.01)	.11
CAD	1.72 (0.55-5.34)	.35
COPD	12.70 (1.41-114.85)	.02
D_lco_	0.96 (0.94-0.98)	<.001
CKD	5.90 (0.69-50.35)	.10
Reduced tolerance to physical activity		
Age	0.96 (0.93-0.99)	.003
ICU admission	2.59 (1.06-6.36)	.04
D_lco_	0.98 (0.96-1.00)	.09

Abbreviations: CAD, coronary artery disease; CKD, chronic kidney disease; COPD, chronic obstructive pulmonary disease; D_lco_, diffusing lung capacity carbon monoxide; ICU, intensive care unit; OR, odds ratio.

^a^ Evaluated using the Short Physical Performance Battery or 2-minute walking test.

When directly questioned, 50 patients (21.0%) reported that their tolerance to exercise had worsened after COVID-19. Univariate analysis (eTable 4 in the Supplement) and logistic regression (Table 3) found that the perception of reduced tolerance to physical exercise was associated with younger age and ICU admission during hospitalization.

### Psychological Symptoms Tests

Finally, we tested patients for PTS symptoms. Results of the IES-R questionnaire were within reference ranges in 136 patients (57.1%), while in 61 patients (25.6%) had mild symptoms, 27 patients (11.3%) had moderate symptoms, and 14 patients (5.9%) had severe symptoms. Male sex was the only factor independently associated with the presence of moderate to severe PTS symptoms (eTable 5 in the Supplement; Table 4).

**Table 4.** Logistic Regression Analysis of Factors Associated With Posttraumatic Stress Symptoms

Factor	OR (95% CI)	*P* value
Sex	0.34 (0.14-0.84)	.02
Modality of oxygen delivery	0.79 (0.50-1.22)	.29
D_lco_	0.97 (0.95-1.00)	.07
Residual dyspnea	2.40 (0.63-9.16)	.20
Chronic kidney disease	3.08 (0.90-10.57)	.07

Abbreviations: D_lco_, diffusing lung capacity carbon monoxide; OR, odds ratio.

## Discussion

Little is known about the lasting effects of SARS-CoV-2 infection in survivors of COVID-19. In this cohort study, we found that a significant proportion of survivors of COVID-19 experienced respiratory or functional impairment 4 months after hospital discharge, with clinically relevant psychological consequences. Indeed, at the end of follow-up, more than half of the study population still had D_lco_ less than 80% of expected. When a more stringent threshold of less than 60% of expected was applied, the proportion of patients with severe impairment decreased to 15%. Thus, a significantly impaired diffusion persisted in a sizable proportion of survivors of COVID-19. The lack of a pre–COVID-19 measurement prevents exact quantification of the association of COVID-19 with PFT deterioration. However, the isolated reduction of D_lco_ with a preservation of other PFT parameters is consistent with damage associated with COVID-19, given that D_lco_ reduction is the most common functional alteration reported in patients with COVID-19.^8^ In line with our results, a study by Huang et al^22^ reported that more than 50% of patients had D_lco_ less than 80% of expected 30 days after hospital discharge. However, a study by Zhao et al^23^ reported only 9 of 55 patients (16.4%) had a D_lco_ less than 80% of expected 3 months after hospital discharge. The populations these studies described were significantly different from ours, being younger (median age, 48 years) and with a very low prevalence of smokers; accordingly, these studies included no patients with underlying pulmonary disease.

As for factors associated with reduced D_lco_, female sex was a significant factor, possibly reflecting fitness level.^24^ Interestingly, a history of CKD and the modality of oxygen administration during hospitalization were associated with reductions in D_lco_, probably owing to a more severe acute illness.^25,26^ Conversely, COPD and ICU admission emerged as factors associated with severe lung function impairment. A reduction of D_lco_ is associated with pulmonary fibrosis in different clinical settings, such as interstitial lung diseases and systemic sclerosis^27,28^; whether survivors of COVID-19 with impaired D_lco_ are at increased risk of progressive lung fibrosis will require a longer follow-up. Indeed, fibrotic evolution has been described after SARS pneumonia.^29^

Some degree of motor impairment was observed in 53.8% of our study population. Different factors might be invoked to explain this observation, including lung damage, circulatory limitation, muscle weakness, critical illness neuropathy, and myopathy. Our data suggest an association of pulmonary function impairment; indeed, D_lco_ and a history of COPD were independently associated with impaired physical function.

Interestingly, many people perceived that COVID-19 had a detrimental impact on their physical performances, and ICU admission being associated with this perception may be associated with deconditioning. Remarkably, age was not associated with reduced D_lco_ or impaired motor function. In COVID-19, the case fatality rate increases with age^30^; therefore, it would be reasonable to expect a higher burden of residual impairment in older patients. Our data are in contrast with this hypothesis. We speculate that older people may have a higher baseline comorbidity burden, which was detrimentally associated with their survival probability during acute illness, but in survivors, the residual damage was not worse than in younger people. Essentially, this finding confirms that older individuals who survive COVID-19 may not be less able than their younger counterparts to revert to their previous state of health, with no accrual of morbidity. This observation has important implications, given that advancing age is often among the major limitations to admit patients with COVID-19 to an ICU.^31^

With regard to psychological health, clinically relevant PTS symptoms were observed in 17% of patients, in line with other studies, such as that by Chang and Park^32^ that reported that approximately 20% of survivors of COVID-19 developed posttraumatic stress disorder. In our data, male sex was the only independent factor associated with PTS symptoms.

Additionally, our data suggest a residual mortality in the first months after discharge from a hospital dedicated to acute care for patients with COVID-19; indeed, around 5% of patients who were discharged after COVID-19 treatment died within a few weeks after discharge. Although we have no information on the causes of death for these patients, this proportion is not negligible and represents, to our knowledge, a worrying novelty.

Finally, we investigated the presence of residual symptoms of disease: dyspnea persisted in approximately 10% of patients who reported experiencing it during the acute phase of COVID-19. Carfi et al^33^ have reported a residual dyspnea in approximately 40% of survivors of COVID-19. However, our study spans a longer follow-up period (4 months vs 2 months). On the other hand, the proportion of patients experiencing chemosensory dysfunction was relatively high. According to a metanalysis by Hajikhani et al,^34^ at presentation, the estimated rate of taste disorders was 49.0% and of olfactory disorders was 61.0%. Data regarding the short-term recovery from these symptoms are divergent. While a study by Iannuzzi et al^35^ reported a complete recovery within 1 month, a study by Locatello et al^36^ reported the persistence of partial or complete chemosensory loss in approximately 30% to 40% of patients. In our study, alterations of taste and smell were still present at 4 months in approximately 17% of patients who had alterations in the acute phase of COVID-19. How long this loss will persist is unknown. Interestingly, one-third of patients reporting arthralgia and myalgia during the acute phase still experienced these symptoms at 4 months. Our findings are in line with 2 cohort studies.^33,37^ However, the proportion of patients who were symptomatic in our study was lower than those reported in the studies by Carfi et al^33^ and Carvalho-Schneidfer et al,^37^ suggesting a progressive improvement over time. Overall, these findings suggest that many patients experience a slow recovery after the acute phase of COVID-19.

### Limitations

This study has several limitations. A key limitation of our study is patient selection: we contacted only patients who had a severe enough COVID-19 to be admitted to a hospital. Moreover, many discharged patients declined study participation for reasons that may have included perceived full recovery in some or the inability or unwillingness to attend extra visits. This might have generated a selection bias, considering that the real proportion of patients still experiencing functional or psychological sequelae might have been lower if all had participated. Our psychological evaluation was limited to PTS symptoms, which does not allow us to draw definitive conclusions about the full psychological impact of COVID-19, which may include many other aspects, such as sleep disturbances, anxiety, and depression. Additionally, we reported an unexpectedly high residual mortality in the first few months after hospital discharge. This is a novel, worrisome finding that warrants further examination.

## Conclusions

In this cohort study, a significant proportion of patients hospitalized for COVID-19 still reported a high proportion of symptoms associated with COVID-19 up to 4 months after hospital discharge, with reduced exercise tolerance being the most common. Other midterm sequelae of COVID-19, such as respiratory and physical functional impairment, may impact psychological health. Residual lung injury may be associated with reduced quality of life in survivors of COVID-19. Although age is a major factor associated with COVID-19–related mortality, 4 months after hospital discharge, there was not a higher residual symptomatic burden in the older patients in this study.

## Notes

Supplement. **eTable 1.** Univariate Analysis for D_LCO_ Less Than 80% **eTable 2.** Univariate Analysis for D_LCO_ Less Than 60% **eTable 3.** Univariate Analysis for Functional Impairment **eTable 4.** Univariate Analysis for Reduced Tolerance to Physical Activity **eTable 5.** Univariate Analysis for PTS Symptoms
