# Supplementary material for: Respiratory and Psychophysical Sequelae Among Patients With COVID-19 Four Months After Hospital Discharge
Source: JAMA Netw Open. 2021 Jan 27;4(1):e2036142. doi: 10.1001/jamanetworkopen.2020.36142 (PMC7841464; doi:10.1001/jamanetworkopen.2020.36142)
Supplement: Supplement. — eTable 1. Univariate Analysis for DLCO Less Than 80% eTable 2. Univariate Analysis for DLCO Less Than 60% eTable 3. Univariate Analysis for Functional Impairment eTable 4. Univariate Analysis for Reduced Tolerance to Physical Activity eTable 5. Univariate Analysis for PTS Symptoms [file jamanetwopen-e2036142-s001.pdf]

Supplemental Online Content

Bellan M, Soddu D, Balbo PE, et al. Respiratory and psychophysical sequelae among patients with COVID-19 four months after hospital discharge. *JAMA Netw Open*. 2021;4(1):e2036142. doi:10.1001/jamanetworkopen.2020.36142

eTable 1. Univariate Analysis for D_{LCO} Less Than 80%

eTable 2. Univariate Analysis for D_{LCO} Less Than 60%

eTable 3. Univariate Analysis for Functional Impairment

eTable 4. Univariate Analysis for Reduced Tolerance to Physical Activity

eTable 5. Univariate Analysis for PTS Symptoms

This supplemental material has been provided by the authors to give readers additional information about their work.

© 2021 Bellan M et al. *JAMA Network Open.*

eTable 1. Univariate Analysis for D_{LCO} Less Than 80%

	DLCO < 80% (n/y)	p
Age, years	60 [47-69] / 65 [51-72]	0.02
Male gender	79 (58.5) / 56 (41.5)	<0.001
Obesity	10 (9.4) / 11 (9.7)	1.00
T2DM	12 (11.3) / 20 (17.7)	0.25
COPD	4 (3.8) / 10 (8.8)	0.17
OSAS	2 (1.9) / 2 (1.7)	1.00
Arterial hypertension	42 (39.6) / 47 (41.6)	0.78
Coronary Artery disease	9 (8.5) / 12 (10.6)	0.65
Atrial fibrillation	4 (3.8) / 11 (9.7)	0.11
Chronic Kidney Disease	2 (1.9) / 12 (10.6)	0.01
Smoking (active-former)	14 (13.2) – 27 (25.5) / 10 (8.8) – 42 (37.2)	0.15
Number of comorbidities (two or less – more than two)	24 (22.6) – 26 (24.5) / 29 (25.6) – 37 (32.3)	0.23
ICU admission	8 (7.5) / 20 (17.7)	0.03
Modality of oxygen delivery (nasalcannula or Venturi mask – Non invasive ventilation-intubation)	45 (42.4) – 18 (17.0) – 6 (5.7) / 45 (39.8) – 28 (24.8) – 15 (13.3)	0.04

© 2021 Bellan M et al. *JAMA Network Open.*

eTable 2. Univariate Analysis for D_{LCO} Less Than 60%

	DLCO<60% (n/y)	p
Age	61 [49-69] / 63.5 [54-75]	0.11
Male gender	118 (63.8) / 17 (50.0)	0.18
Obesity	17 (9.2) / 4 (11.8)	0.75
T2DM	24 (13.0) / 8 (23.5)	0.12
COPD	8 (4.3) / 6 (17.6)	0.01
OSAS	3 (1.6) / 1 (2.9)	0.49
Arterial hypertension	74 (40.0) / 15 (44.1)	0.71
Coronary Artery disease	16 (8.6) / 5 (14.7)	0.34
Atrial fibrillation	12 (6.5) / 3 (8.8)	0.71
Chronic Kidney Disease	9 (4.9) / 5 (14.7)	0.05
Smoking (active-former)	22 (11.9) - 54 (29.2) / 2 (5.9) – 15 (44.1)	0.18
Number of comorbidities (two or less – more than two)	45 (24.3) – 49 (26.5) / 8 (23.5) – 14 (41.2)	0.19
ICU admission	15 (8.1) / 13 (38.2)	<0.001
Modality of oxygen delivery (nasalcannula or Venturi mask – Non invasive ventilation-intubation)	77 (41.6) – 37 (20.0) – 12 (6.5) / 13 (38.2) – 9 (26.5) – 9 (26.5.9)	<0.001

© 2021 Bellan M et al. *JAMA Network Open.*

eTable 3. Univariate Analysis for Functional Impairment

	Functional impairment (n/y)	p
Age	60 [47-69] / 65 [53-73]	0.01
Male gender	73 (66.4) / 69 (53.9)	0.06
Obesity	5 (4.5) / 20 (15.6)	0.005
T2DM	12 (10.9) / 24 (18.8)	0.10
COPD	1 (0.9) / 13 (10.1)	0.002
OSAS	1 (0.9) / 4 (3.13)	0.38
Arterial hypertension	41 (37.3) / 57 (44.5)	0.29
Coronary Artery disease	7 (6.4) / 15 (11.7)	0.18
Atrial fibrillation	5 (4.6) / 12 (9.4)	0.21
Chronic Kidney Disease	1 (0.9) / 14 (10.9)	0.001
Smoking (active-former)	12 (10.9) – 35 (31.8) / 13 (10.2) – 39 (30.5)	0.95
Number of comorbidities (two or less – more than two)	21 (19.1) – 20 (18.2) / 37 (28.9) – 48 (37.5)	0.001
ICU admission	9 (8.2) / 19 (14.8)	0.16
Modality of oxygen delivery (nasalcannula or Venturi mask – Non invasive ventilation- intubation)	53 (48.2) – 17 (15.5) – 7 (6.4) / 49 (38.3) – 32 (25.0) – 14 (10.9)	0.12
DLCO	83 [75-93] / 73 [58-84]	0.001

© 2021 Bellan M et al. *JAMA Network Open.*

eTable 4. Univariate Analysis for Reduced Tolerance to Physical Activity

	Functional impairment (n/y)	p
Age	63 [52-72] / 56 [47-66]	0.01
Male gender	110 (58.0) / 32 (64.0)	0.52
Obesity	19 (10.1) / 6 (12.0)	0.79
T2DM	30 (16.0) / 6 (12.0)	0.66
COPD	11 (5.9) / 3 (6.0)	1.00
OSAS	3 (1.6) / 2 (4.0)	0.28
Arterial hypertension	81 (43.1) / 17 (34.0)	0.26
Coronary Artery disease	19 (10.1) / 3 (6.0)	0.58
Atrial fibrillation	14 (7.4) / 3 (6.0)	1.00
Chronic Kidney Disease	10 (5.3) / 5 (10.0)	0.32
Smoking (active-former)	23 (12.2) – 56 (29.8) / 2 (4.0) – 18 (36.0)	0.22
Number of comorbidities (two or less – more than two)	50 (26.6) – 54 (28.7) / 8 (16.0) – 14 (28.0)	0.23
ICU admission	17 (9.0) / 11 (22.0)	0.02
Modality of oxygen delivery (nasalcannula or Venturi mask – Non invasive ventilation-intubation)	82 (43.6) – 40 (21.3) – 13 (6.9) / 20 (40.0) – 9 (18.0) – 8 (16.0)	0.25
DLCO	80 [70-89] / 73 [58-84]	0.04

© 2021 Bellan M et al. *JAMA Network Open.*

eTable 5. Univariate Analysis for PTS Symptoms

	PTS symptoms (n/y)	p
Age	62 [50-71] / 61 [54-70]	0.88
Male gender	114 (57.9) / 28 (68.3)	0.20
Obesity	21 (10.7) / 4 (9.8)	1.00
T2DM	30 (15.2) / 6 (14.7)	1.00
COPD	13 (6.6) / 1 (2.4)	0.47
OSAS	4 (2.0) / 1 (2.4)	1.00
Arterial hypertension	79 (40.1) / 19 (46.3)	0.49
Coronary Artery disease	18 (9.1) / 4 (9.8)	1.00
Atrial fibrillation	15 (7.6) / 2 (4.9)	0.75
Chronic Kidney Disease	9 (4.6) / 6 (14.6)	0.003
Smoking (active-former)	21 (10.7) – 60 (30.5) / 4 (9.8) – 14 (34.1)	0.89
Number of comorbidities (two or less – more than two)	46 (23.3) – 58 (29.4) / 12 (29.3) – 10 (24.4)	0.67
ICU admission	23 (11.7) / 5 (12.2)	1.00
Modality of oxygen delivery (nasalcannula or Venturi mask – Non invasive ventilation-intubation)	81 (41.1) – 46 (23.3) – 16 (8.1) / 21 (51.2) – 3 (7.3) – 5 (12.2)	0.13
DLCO	80 [69-90] / 75 [67-83]	0.13
Dyspnea at presentation	105 (53.3) / 24 (58.5)	0.60
Residual dyspnea	8 (4.0) / 5 (12.2)	0.05

© 2021 Bellan M et al. *JAMA Network Open.*
